# Weakly Supervised Fine-Grained Discrimination of Wheat Mold Using Local RGB–HSI Fusion

**DOI:** 10.3390/foods15122232

**Published:** 2026-06-20

**Authors:** Le Xiao, Shengtong Wang, Lulu Niu

**Affiliations:** 1 Key Laboratory of Grain Information Processing and Control, Henan University of Technology, Ministry of Education, Zhengzhou 450001, China; 2 Henan Key Laboratory of Grain Photoelectric Detection and Control, Henan University of Technology, Zhengzhou 450001, China; 3 Henan Grain Big Data Analysis and Application Engineering Research Center, Henan University of Technology, Zhengzhou 450001, China

**Keywords:** wheat, mold severity, cross-modal, fine-grained, weakly supervised

## Abstract

Wheat is a major staple crop, and storage mold growth poses a severe threat to grain safety and quality stability. Natural mold development in stored wheat exhibits subtle, localized, and highly heterogeneous characteristics. Existing unimodal methods and global fusion approaches generally suffer from insufficient local feature sensitivity, hindering fine-grained mold severity grading. To address this limitation, we propose a Mask-Guided Fine-Grained Fusion Network, a weakly supervised framework based on local RGB–HSI fusion. This framework employs a dynamic parallel A/B experimental design to construct time-matched proxy labels via weakly supervised learning. A standardized preprocessing pipeline including single-kernel extraction, foreground segmentation, and cross-modal registration is established to resolve RGB–HSI spatial misalignment, ensuring physical-level spatial consistency of multimodal features. The model incorporates a Foreground-Aware Spectral Recalibration (FASR) module to suppress background noise, a Mask-Guided Dilated Cross-modal Local Attention (MDCLA) mechanism to establish fine-grained local mappings between RGB visual phenotypes and hyperspectral responses, and a sample-level adaptive fusion strategy to dynamically weight features by modal reliability, enhancing representation of complex samples across all mold stages. Experiments show that the Mask-Guided Fine-Grained Fusion Network achieves 0.9689 classification accuracy, 0.9698 Macro-F1 score, and 0.0593 Mean Absolute Error (MAE), significantly outperforming state-of-the-art unimodal deep models and global attention fusion baselines. This work provides a proof-of-principle framework for fine-grained non-destructive mold risk assessment in stored wheat.

## 1. Introduction

Nutritional deterioration and mycotoxin accumulation induced by wheat mold pose severe threats to grain storage and food safety [1,2]. In practical storage environments, wheat mold is driven by complex microenvironments rather than being induced by a single pathogen; it manifests as a continuous natural process characterized by the asynchronous evolution of external phenotypes and internal compositions [3,4]. This fundamental characteristic makes it reasonable to formulate a fine-grained classification task oriented toward progressive risk evolution [5]. Traditional destructive physicochemical analyses are inadequate to meet the requirements for rapid and non-destructive detection in grain storage scenarios [6]. In the realm of non-destructive sensing, machine vision excels at capturing external color and texture [7], whereas hyperspectral imaging (HSI) demonstrates immense potential by reflecting internal biochemical and spectral responses [8,9,10]. Nevertheless, single-modality information exhibits inherent limitations in fine-grained recognition tasks: visible imaging may be insufficient for capturing latent internal variations, while hyperspectral data lacks sufficient local spatial cues. To address the limitations of unimodal imaging technologies, fusing multi-source information from microscopic RGB and hyperspectral images has emerged as a crucial direction for enhancing the capability of precise mold recognition [11,12].

Fungal colonization in stored wheat kernels is not an abrupt, spatially uniform event. It progresses gradually under local microenvironmental conditions including moisture accumulation, temperature variation, oxygen availability, and kernel-to-kernel contact [13]. Fungal propagules initially adhere to the pericarp, germ region, crease, or mechanically damaged micro-sites, followed by localized hyphal growth and gradual invasion into the kernel surface and internal tissues. Mold development at the single-kernel scale exhibits spatial heterogeneity, asynchronous progression, and weak early phenotypic expression. Visible changes in kernel color, texture, and surface morphology are often subtle during adjacent mold stages, particularly during transitions from safe to critical or critical to hazardous status, and are partially masked by natural variations among kernels. Purely RGB-based discrimination is unreliable for fine-grained severity grading [14]. Hyperspectral imaging in the 902–1744 nm range captures biochemical and physiological perturbations associated with fungal growth before or during the appearance of obvious visual symptoms. Mold development alters the water state and distribution inside kernels, accelerates carbohydrate and lipid degradation, and induces changes in protein- and cell-wall-related constituents. These processes are directly related to absorption features primarily arising from overtones and combination vibrations of O–H, C–H, and N–H bonds within the selected spectral range [15]. RGB images provide local phenotypic cues, while HSI reflects internal compositional responses. The asynchronous and complementary nature of these two modalities forms the biological basis for local RGB–HSI fusion in fine-grained wheat mold discrimination. At the same time, fully supervised kernel-level labeling is difficult in this task. Once a kernel is subjected to destructive microbiological testing, such as fungal spore counting, it can no longer be used for continuous RGB–HSI. Replacing it with another kernel would break the one-to-one correspondence between imaging features and microbiological status, since fungal loads vary among kernels even under the same incubation condition. Therefore, direct kernel-level annotation is impractical for dynamic mold monitoring, making it necessary to introduce a weakly supervised strategy based on time-matched proxy labels.

Regarding visible light image analysis, the color, texture, and morphological alterations induced by fungal infection in wheat kernels can be utilized to distinguish between healthy and damaged samples. Abbaspour-Gilandeh et al. extracted 21 apparent texture features, achieving high-accuracy differentiation of healthy and infected kernels [16]. Jirsa and Polišenská indicated that color parameters are more sensitive for identifying healthy and damaged kernels [17]. Ropelewska et al. demonstrated that in the binary classification task of healthy and infected kernels, texture features extracted from color images could achieve classification accuracies of 92–100% and 95–100% under specific settings [18]. These results suggest that visible light imaging exhibits strong practicality when external symptoms are pronounced; however, it relies predominantly on visible phenotypes and responds inadequately to the latent changes of mold and the subtle discrepancies between adjacent severity grades, making it difficult to independently support fine-grained classification during the continuous molding process.

Hyperspectral imaging (HSI) enables the simultaneous acquisition of spatial structures and continuous spectral reflectance information, rendering it highly sensitive to internal compositional perturbations, fungal infection, and mycotoxin-related alterations within kernels. Alisaac et al. demonstrated significant correlations between hyperspectral signals and both the fungal infection status and mycotoxin contamination in wheat kernels and flour [19]. Through the integration of Vis-NIR HSI with microscopy and SR-FTIR microspectroscopy, Yao et al. validated the potential of HSI for the non-destructive characterization of microscopic discrepancies related to deoxynivalenol (DON) [10]. Povolotckaia et al. utilized Principal Component Analysis (PCA) to differentiate spectral profiles induced by various Fusarium species [20]. Furthermore, Yang et al. advanced this research to the level of severity grading, demonstrating that the hyperspectral approach possesses the requisite capability for classification tasks [21]. However, as pointed out by Rogers et al., hyperspectral modeling generally grapples with challenges such as band redundancy and inconsistent feature selection strategies [22]. Consequently, its application in the fine-grained differentiation of adjacent grades during continuous molding processes remains to be further optimized.

With the advancement of fusion methodologies, Huang et al. showed that in diagnosing the severity of Fusarium head blight at the ear level, a PSO-SVM model fusing spectral and color features outperformed single-feature models [23]. From the perspective of kernel-level multispectral and hyperspectral imaging, Delwiche et al. noted that the definition of the region of interest (ROI) significantly affects kernel-level recognition performance and modeling stability [24]. This underscores the complementarity between RGB phenotypic information and spectral information. Multimodal hyperspectral imaging has shown clear advantages in agricultural quality assessment by combining external RGB phenotypes with internal spectral responses. RGB images provide color, texture, and morphological information, whereas HSI captures biochemical variations related to moisture redistribution, substrate degradation, and fungal activity. Deep fusion, cross-attention, and Transformer-based methods further improve the modeling of complementary visual–spectral features. For single-kernel wheat mold grading, existing fusion strategies still have limitations. Many methods rely on feature concatenation, global aggregation, or unconstrained token-level attention, which are mainly suited to fully supervised or scene-level tasks. Wheat mold cues are weak, heterogeneous, and spatially localized, often appearing around the germ, crease, ventral groove, or damaged surface, while most kernel regions remain visually normal. Global attention may introduce interference from healthy tissue, contours, shadows, or residual background pixels, weakening subtle RGB–HSI correlations related to mold development. The proposed MDCLA addresses this mismatch by constraining cross-modal interaction to local foreground neighborhoods. Unlike conventional cross-attention, it does not calculate correlations over the entire feature map. Unlike Transformer-based fusion, it does not rely on global token-to-token interaction. Instead, RGB features guide the selection of local hyperspectral responses under mask constraints, allowing the model to focus on lesion-related visual–spectral correspondences. This local design is more consistent with the spatially heterogeneous nature of wheat mold progression and is expected to improve fine-grained discrimination between adjacent mold severity grades.

In summary, existing research addressing wheat mold discrimination still exhibits several critical shortcomings: (1) Insufficient focus on fine-grained differentiation: Most current studies focus on binary contamination screening or macroscopic severity assessment, with limited investigation into the differentiation of adjacent severity grades during the continuous molding process at a single-kernel scale [24,25]. (2) Inadequate systematic research on multimodal data alignment: Systematic investigations into the construction of paired micro-RGB–HSI datasets and their cross-modal spatial correspondences for individual kernels remain insufficient [22,24]. (3) Limited exploitation of local constraints in fusion methodologies: Existing fusion methods make limited use of local spatial correspondences and foreground region constraints, which renders them inadequate for meeting the rigorous requirements of fine-grained classification tasks in natural molding scenarios [23].

To address existing deficiencies in single-kernel fine-grained mold discrimination, cross-modal RGB–HSI spatial alignment, and effective multimodal fusion, the primary contributions of this paper are as follows: (1) Construction of a weakly supervised single-kernel RGB–HSI paired dataset for wheat mold risk grading. A dynamic parallel A/B design was used to resolve the conflict between continuous non-destructive imaging and destructive microbiological annotation. Time-matched microbiological results from Group B were transferred to Group A to generate proxy labels for weakly supervised learning. (2) Establishment of a multimodal preprocessing pipeline tailored for single-kernel wheat. Addressing the scale and modal discrepancies inherent in microscopic imaging, a spatial registration scheme was designed to achieve precise spatial alignment between external phenotypes and internal spectral profiles, complemented by foreground region constraints. (3) Proposal of the Mask-Guided Fine-Grained Fusion Network (MFGF-Net). This network integrates foreground-aware spectral recalibration, local cross-modal attention, and sample-level adaptive fusion to substantially reinforce the complementary expression between RGB phenotypic features and HSI spectral responses.

## 2. Materials and Methods

To address subtle inter-grade mold discrepancies, unimodal limitations, and individual kernel annotation challenges, we establish a microscopic RGB–HSI paired acquisition pipeline and construct severity labels via parallel-group sampling. Furthermore, a local cross-modal fusion model is proposed for fine-grained mold discrimination by integrating complementary RGB phenotypic and HSI spectral features.

### 2.1. Sample Preparation

In this study, four wheat cultivars were used, namely Zhengmai 136 (ZM136), Zhongmai 69 (ZM69), Zhengmai 139 (ZM139), and Zhengmai 918 (ZM918). All experimental grain samples were harvested in June 2025 from Henan Province, China. In the microbiological reference group synchronously established for the wheat samples in this study, high-throughput sequencing and colony morphological analysis revealed that the natural microflora associated with grain spoilage was dominated by typical storage mold genera, specifically *Aspergillus* spp., including *A. flavus* and *A. niger*, and *Penicillium* spp., accompanied by minor field residuals of Fusarium spp. This multi-variety sample matrix covers inter-cultivar variability and provides a useful foundation for evaluating the generalization performance of the proposed fine-grained discrimination network.

A screened 200 g healthy wheat sample with an initial moisture content of 12.6% was incubated at 30 ± 1 °C and 85 ± 2% RH. To resolve the fundamental conflict between continuous non-destructive imaging and destructive microbiological assays, the sample was partitioned into two groups. 400 intact kernels with a total mass of 21.4 g were selected as Group A for repeated non-destructive RGB–HSI. The remaining 178.6 g of wheat was assigned to Group B as an independent destructive microbiological reference pool. Before incubation, Group B was pre-divided into thirteen independent 10 g subsamples and an unused reserve portion of 48.6 g. Each 10 g subsample was placed in a separate container and incubated in parallel under the same environmental conditions as Group A. To reduce local clustering and excessive kernel-to-kernel contact, the kernels in each subsample were spread as a thin layer with minimal overlap as far as practically possible. At each incubation time point from Day 0 to Day 12, one pre-assigned 10 g subsample was removed and subjected to destructive microbiological analysis. Therefore, Group B provided 13 time-matched microbiological reference samples in total, corresponding to a cumulative destructive sampling mass of 130 g. The remaining 48.6 g of Group B wheat was kept as unused reserve material and was not included in proxy-label generation or model training.

**Figure 1:**
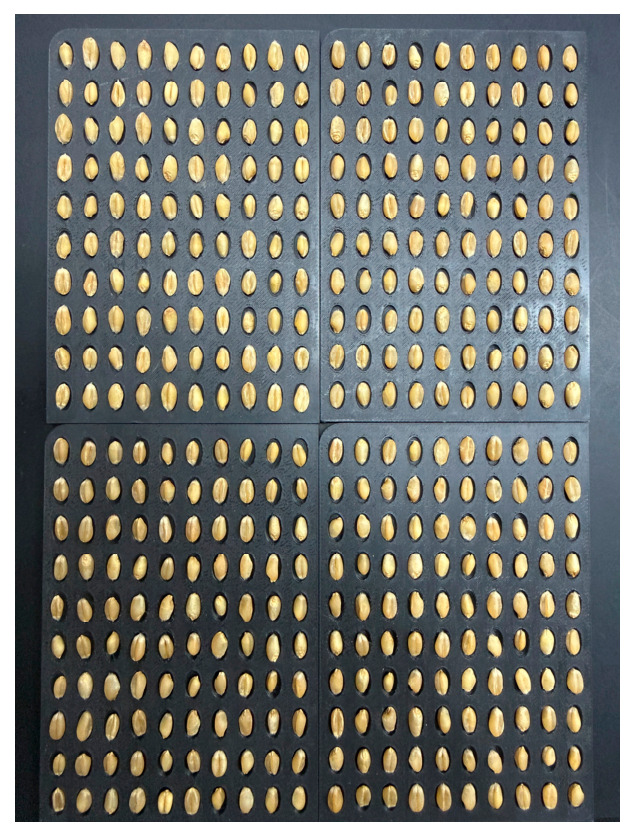
Physical arrangement of the 400 Group A wheat kernels. The kernels were placed on four fixed black positioning plates arranged in a 2 × 2 layout. Each plate had physical dimensions of 10.5 cm × 7.2 cm and contained a 10 × 10 grid of independent kernel slots.

Group A contained 400 fixed kernels and was monitored daily from Day 0 to Day 12 using microscopic RGB (BETICAL SZ7D-T510, Evident Optical Technology (Guangzhou) Co., Ltd., Guangzhou, China; 2592 × 1944 pixels) and HSI (GaiaSorter-RT17, Jiangsu Dualix Spectral Imaging Technology Co., Ltd., Wuxi, China; 902–1744 nm, 5 nm resolution) imaging under constant geometry. Hyperspectral data were acquired at each incubation time point by scanning the entire fixed array containing all 400 Group A kernels as a single hyperspectral scene under identical imaging geometry. Kernel-level hyperspectral samples were subsequently derived from the whole-scene image through foreground segmentation, connected-component analysis, centroid-based ordering, and single-kernel cropping. Accordingly, each hyperspectral acquisition covered all 400 Group A kernels, yielding 400 kernel-level HSI samples per day and a total of 5200 kernel-level HSI observations over the 13-day incubation period. For Group A, the 400 wheat kernels were individually placed on four black positioning plates arranged in a 2 × 2 layout, with each plate containing a 10 × 10 grid of independent kernel slots. Each kernel occupied a fixed slot throughout the incubation period. This physical arrangement ensured that the same kernels could be repeatedly imaged from Day 0 to Day 12 and consistently matched during single-kernel cropping, time-series tracking, and RGB–HSI cross-modal pairing. A representative photograph of the kernel arrangement is shown in Figure 1. To support time-aligned sample organization, each Group A kernel was assigned a unique identification key and fixed spatial coordinate, enabling time-series tracking, single-kernel cropping, and RGB–HSI cross-modal pairing.

Group B samples underwent rapid HSI scanning prior to destructive testing to verify data synchronization between the two groups. Microbiological analysis was performed using the spore counting method in accordance with the LS/T 6132-2018 standard [26]. Fungal spore counts were classified into four grades based on national grain storage safety standards: <$1.0\;\times \;10^{5},\;1.0\times 10^{5}–9.9\times 10^{5},\;1.0\times 10^{6}–9.9\times 10^{6},$ and ≥1.0 × 10^7^ spores/g, corresponding to four grain storage risk levels: Safe, Critical, Hazardous, and Severe [23]. In this study, these risk levels were mapped to the wheat mold development process, resulting in four distinct mold stages: Days 0–3 (Safe stage), Days 4–7 (Critical stage), Days 8–10 (Hazardous stage), and Days 11–12 (Severe stage). Consequently, the mold severity label for each Group A sample was assigned based on the microbiological test results of Group B at the same incubation time point. Weak proxy labels were obtained through label transfer from synchronized Group B reference measurements, thereby establishing a single-kernel multimodal weakly supervised dataset based on the parallel-group reference method [25].

### 2.2. Construction and Preprocessing of the Multimodal Paired Dataset

#### 2.2.1. Single-Kernel Extraction and Sequential Pairing

**Figure 2:**
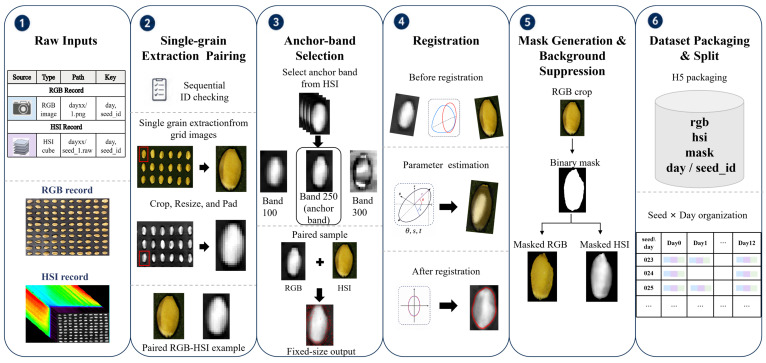
Construction and preprocessing workflow for RGB–HSI paired samples. The workflow consists of six core steps: single-kernel extraction, foreground segmentation, size normalization, cross-modal registration, foreground-mask generation, and HDF5-based dataset packaging. Arrows indicate the sequential preprocessing flow. RGB thumbnails represent visible-light images, whereas the rainbow-colored cube represents the hyperspectral image cube. The white and gray binary masks correspond to the foreground and background regions of the sample kernel, respectively. In the registration module, the colored contours indicate the cross-modal sample-kernel outlines used to evaluate registration performance. In the dataset-packaging module, the colored blocks represent the organization and packaging status of different data fields, and ellipses indicate repeated data entries omitted due to space limitations.

Utilizing individual wheat kernels as analytical units effectively captures the spatial heterogeneity of mold spoilage, which typically initiates in localized microenvironments during storage. Because batch-averaged spectra obscure signals from abnormal kernels, single kernels were adopted as both model inputs and prediction units. To ensure data consistency for subsequent modeling, a standardized preprocessing workflow (Figure 2) was established, comprising single-kernel extraction, foreground segmentation, cross-modal registration, size normalization, and unified packaging.

For RGB images, kernels were arranged in a fixed array with unique identifiers and stable spatial positions. Single-kernel cropping was performed utilizing predefined array coordinates, and each kernel was uniformly saved as a 384 × 256-pixel patch to preserve surface texture, edge contours, and local morphological details.

For hyperspectral images, raw data were initially corrected via white-dark reference calibration. A reference band centered at 1321 nm (Band 250) was selected due to its high kernel-background contrast. Binary segmentation was executed using Otsu’s automatic thresholding, followed by morphological closing with a 3 × 3 structuring element and connected-component filtering to eliminate holes and spurious targets, thereby yielding stable single-kernel foreground regions.

Following segmentation, kernels were sorted by centroid coordinates to establish a one-to-one correspondence with the RGB array, enabling accurate cross-modal pairing. Finally, each hyperspectral kernel was cropped utilizing the minimum bounding box of its connected component, augmented with a 6-pixel outward margin to preserve contour and edge context.

#### 2.2.2. Size Normalization and Cross-Modal Registration of Samples

Following extraction and identifier matching, cross-modal paired samples were constructed. To mitigate discrepancies in spatial resolution, field of view, and boundary morphology, size normalization was performed prior to feature interaction. Hyperspectral patches initially sized at 96 × 64 pixels were resampled to match the 384 × 256 pixel dimensions of the RGB images. Bicubic and area interpolation methods were applied to the hyperspectral and RGB data, respectively, ensuring spatial consistency without compromising textural or structural details.

To establish spatial correspondence, both the RGB image and a selected hyperspectral reference band were converted to normalized grayscale formats. An enhanced correlation coefficient (ECC) algorithm was subsequently employed to estimate the cross-modal geometric transformation matrix. Given the minor translation, rotation, and scaling variations between modalities, an affine transformation model was prioritized. For samples where affine convergence failed, a fallback translation model was utilized to re-estimate parameters, thereby ensuring the robustness of the registration pipeline.

The derived transformation matrix was applied band-wise to the hyperspectral data cube, aligning the HSI data with the RGB spatial domain. This unified coordinate system facilitates subsequent local cross-modal interactions. Unlike direct global concatenation, this precise registration strategy allows the model to jointly exploit local RGB phenotypic cues and continuous HSI spectral responses within accurately matched foreground regions.

#### 2.2.3. Foreground-Mask Generation, Normalization, and HDF5 Packaging

After cross-modal spatial registration, a single-kernel foreground mask was generated from foreground segmentation results and mapped to the unified spatial coordinate system, serving as an external spatial constraint input for the subsequent model. The foreground mask, denoted as $M\;\in \;\{0,\;1{\}}^{H\times W}$, was uniformly mapped to the range [0, 1]. To mitigate inter-sample variations in brightness and reflectance scales, all input modalities—comprising the RGB images, hyperspectral cubes, and the foreground mask—were numerically normalized to a [0, 1] range. Finally, the preprocessed data were packaged into an HDF5 database. Each paired sample integrated the registered RGB image, HSI cube, foreground mask, mold severity label, sample identifier, and incubation time point, ensuring consistent data loading for network training.

The registered RGB image and hyperspectral cube were resized to 384 × 256 × 3 and 384 × 256 × B, respectively, where B denotes the number of retained hyperspectral bands after preprocessing. In this study, B was set to 512, covering the spectral range of 902–1744 nm. The foreground mask was resized to the same spatial resolution and used as a spatial constraint rather than being directly concatenated with the RGB or HSI input.

In the HSI branch, a 1 × 1 convolution was first used to project the B-band hyperspectral input to a unified feature dimension of C = 32. The projected feature map was then downsampled by a 3 × 3 convolution with a stride of 2, followed by three residual blocks. Each residual block consisted of two 3 × 3 convolutional layers, batch normalization, ReLU activation, and an identity shortcut. The RGB branch used the same feature dimension and consisted of an initial 3 × 3 convolution with a stride of 2 followed by two residual blocks, ensuring dimensional consistency for cross-modal interaction.

The MDCLA module used RGB features as queries and HSI features as keys and values. Multi-head attention was adopted with four heads, and the channel dimension of each head was 8. The three local interaction stages used 3 × 3, dilated 5 × 5 with a dilation rate of 2, and 3 × 3 attention windows, respectively. A 3 × 3 convolutional refinement layer was added after each interaction stage to stabilize the fused representation.

For sample-level adaptive fusion, mask-constrained global average pooling was applied to the RGB, HSI, and cross-modal feature maps, producing three C-dimensional descriptors. These descriptors were concatenated and fed into a two-layer gating network with a hidden dimension of C. The gating network consisted of a fully connected layer, ReLU activation, dropout with a probability of 0.3, and a final fully connected layer followed by Softmax normalization to generate three adaptive weights. The classifier consisted of mask-constrained global average pooling, a 32-unit fully connected layer, ReLU activation, dropout with a probability of 0.3, and a four-neuron output layer corresponding to the four mold severity grades.

### 2.3. Multimodal Model for Discriminating Wheat Mold Severity Grading

**Figure 3:**
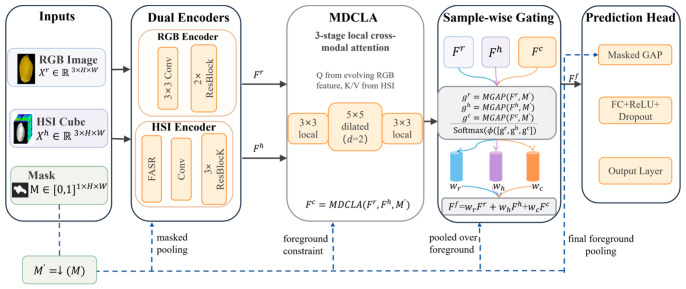
Overall architecture of MFGF-Net. The network takes registered RGB images, HSI cubes, and foreground masks as inputs and consists of four stages: dual-branch feature extraction, mask-constrained local cross-modal interaction, sample-level adaptive fusion, and mold severity prediction. Black solid arrows indicate the forward data and feature flow between network modules. Blue dashed arrows indicate the mask-guidance paths, showing how the foreground mask is mapped or downsampled and then applied to the FASR, MDCLA, sample-wise gating, and final masked global average pooling modules.

To leverage the complementary spatial phenotypes and spectral responses of the paired modalities, this study proposes MFGF-Net for mold severity grading. The architecture integrates the registered RGB image, HSI cube, and foreground mask. Guided by a unified foreground constraint, MFGF-Net executes fine-grained recognition via local cross-modal interaction and sample-level adaptive fusion, as shown in Figure 3.

#### 2.3.1. Overall Network Architecture

Let the input RGB image be denoted as $X^{r}\;\in {\;R}^{3\times H\times W}$ and the hyperspectral image as $X^{h}\;\in \;R^{B\times H\times W}$, where B denotes the number of hyperspectral bands, and H and W denote the spatial height and width, respectively. The foreground mask is denoted as $M\;\in \;\{0,\;1{\}}^{1\times H\times W}$. As established during preprocessing, both modalities share identical spatial dimensions to facilitate cross-modal interaction.

The MFGF-Net architecture consists of four core components: the Foreground-Aware Spectral Recalibration (FASR) module, a dual-branch RGB–HSI encoder, the Mask-Guided Dilated Cross-Modal Local Attention (MDCLA) module, and a sample-level adaptive fusion head. First, the FASR module uses a foreground mask to recalibrate spectral bands, suppressing background noise in channel-wise statistics. Then, the parallel RGB and HSI branches extract spatial phenotypic features and spectral-spatial joint features, respectively, which undergo local cross-modal interaction within the foreground neighborhood through the MDCLA module. Finally, the adaptive fusion head dynamically integrates unimodal and cross-modal features to output the final spoilage prediction result.

#### 2.3.2. Foreground-Aware Spectral Recalibration Module (FASR)

Hyperspectral images have high dimensions and significant band redundancy. Even after preprocessing, residual background edges and local shadows can still interfere with channel-wise statistics. If band weights are estimated directly from the entire image, background noise will inevitably be introduced, which in turn weakens the discriminative signals of mold-affected regions. Therefore, we place the FASR module at the front end of the HSI branch, strictly limiting the spectral recalibration operation to the foreground regions.

Let the b band of the input hyperspectral image $X^{h}$ be considered. Mask-constrained global average pooling was applied using the foreground mask to obtain the corresponding foreground statistic:

(1) $$S_{b}\;=\;\frac{\sum_{i=1}^{H}\sum_{j=1}^{W}⊙M_{\mathrm{ij}}X_{b,\mathrm{ij}}^{h}}{\sum_{i=1}^{H}\sum_{j=1}^{W}⊙M_{\mathrm{ij}}+\varepsilon },$$

where $S_{b}$ denotes the average response of the b band within the foreground region, $M_{i,j}$ is the mask value at position (i, j), and ε is a small constant used to prevent division by zero. The statistics of all bands are then assembled into a vector $s\;\in \;R^{B}$, which is fed into a channel-recalibration network consisting of two fully connected mappings to obtain the band-weight vector $w\;\in \;[0,\;1{]}^{B}$:

(2) $$w\;=\;\sigma \left(W_{2}\delta (W_{1}s)\right),$$

where $W_{1}$ and $W_{2}$ denote the two fully connected transformations, δ(·) denotes the nonlinear activation function, and σ(·) denotes the Sigmoid activation function. The resulting band weights are finally applied to the original hyperspectral input:

(3) $${\tilde{X}}_{b,i,j}^{h}\;={\;w}_{b}\cdot X_{b,i,j}^{h},$$

By estimating channel-wise statistics from foreground regions, the module reduces the influence of background areas during band-weight estimation. As a result, the subsequent HSI branch can focus more effectively on informative spectral bands associated with mold spoilage.

#### 2.3.3. RGB–HSI Dual-Branch Feature Encoding

After completing spectral recalibration, the RGB and HSI modalities are processed using separate feature encoding branches to extract complementary mid-to-high-level feature representations. The RGB branch extracts spatial phenotypic features including color, texture, and local morphology; the HSI branch captures spectral reflectance variations and their local structural associations.

In the HSI branch, a 1 × 1 convolution is first used to map the original hyperspectral input ${\tilde{X}}^{h}$ from B bands to a unified feature dimension C, reducing the number of parameters and facilitating subsequent cross-modal interaction. A 3 × 3 convolution with a stride of 2 is then applied for initial downsampling, followed by cascaded residual blocks to extract high-level joint spectral–spatial features. The resulting feature map is denoted as $F^{h}\;\in \;R^{C\times H^{\prime }\times W^{\prime }}$.

Parallelly, the input image $X^{r}$ is passed through a 3 × 3 convolution with a stride of 2 and a nonlinear activation function for shallow feature mapping. Two residual blocks are then cascaded to further extract surface color, textural perturbations, and microscopic morphological differences, yielding the RGB feature representation $F^{r}\;\in {\;R}^{C\times H^{\prime }\times W^{\prime }}$. These features provide spatial-structure and texture-based query cues for subsequent cross-modal interaction.

#### 2.3.4. Mask-Guided Dilated Cross-Modal Local Attention Module (MDCLA)

**Figure 4:**
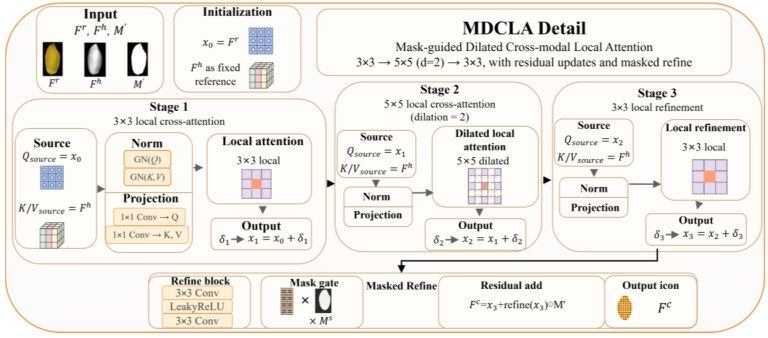
Schematic structure of the MDCLA module. RGB features are used as queries, while HSI features are used as keys and values. Under foreground-mask constraints, the module performs three-stage local cross-modal attention to obtain fine-grained fused representations.

Traditional multimodal models that rely on global feature concatenation or full-image attention mechanisms struggle to achieve ideal results in mold severity grading tasks. Global fusion inherently carries the risk of weak lesion signals being overwhelmed by dominant background or contour features, while unconstrained cross-modal attention can exacerbate spatial mismatches caused by residual registration errors. To overcome these limitations, the Mask-guided Dilated Cross-Modal Local Attention (MDCLA) module strictly confines feature interaction within the foreground neighborhood. This study designed an MDCLA module. As shown in Figure 4, MDCLA uses the RGB feature $F^{r}$ as the Query and the HSI feature $F^{h}$ as the Key and Value. Cross-modal attention is computed only within local foreground neighborhoods, preventing irrelevant background regions from participating in intermodal information exchange.

Let $q_{p}$ denote the RGB query vector at position p, and let its local neighborhood window be denoted as N(p). For any position u ∈ N(p), the corresponding HSI key and value vectors are denoted as $k_{u}$ and $v_{u}$, respectively. The local cross-modal attention weight from position p to neighboring position u is then defined as:

(4) $$\alpha_{p,u}=\frac{\exp\left(\frac{q_{p}^{⊤}k_{u}}{\sqrt{d}}\right)\cdot M_{u}}{\sum_{v\in N(p)}\exp\left(\frac{q_{p}^{⊤}k_{v}}{\sqrt{d}}\right)\cdot M_{v}},$$

where d denotes the single-head feature dimension, and $M_{u}$ denotes the foreground-mask value at position u. Using this weight to compute the weighted sum of the HSI value vectors within the neighborhood yields the cross-modal interaction feature at position p:

(5) $$f_{p}^{c}\;=\;\sum_{u\in N(p)}\alpha_{p,u}v_{u},$$

To balance local details with mid-scale contextual information, MDCLA adopts a three-stage local interaction structure. The first stage uses a 3 × 3 local window to establish basic neighborhood interactions. The second stage employs a 5 × 5 dilated window with a dilation rate of 2 to expand the receptive field. The third stage again uses a 3 × 3 local window to refine and update the features. A convolutional refinement unit can be appended after each interaction stage to improve the stability of the local fusion results.

#### 2.3.5. Sample-Level Adaptive Fusion

After local cross-modal interaction, the network obtains the RGB-branch feature $F^{r}$, the HSI-branch feature ${\;F}^{h}$, and the cross-modal interaction feature $F^{c}$ output by MDCLA. Because different samples may vary in the salience of appearance textures, the strength of spectral responses, and the reliability of each modality, this study further introduces a sample-level adaptive fusion module to dynamically integrate the three feature streams with learned weights. Mask-constrained global average pooling is applied to $F^{r},\;F^{h}$ and $F^{c}$, yielding the corresponding sample-level descriptor vectors $g^{r},\;g^{h}$ and $g^{c}$. Taking the RGB branch as an example,

(6) $$g^{r}=MGAP\left(F^{r},M^{\prime }\right)\;,$$

where MGAP denotes mask-constrained global average pooling, and $M^{\prime }$ denotes the foreground mask downsampled to the feature scale. The HSI branch and the interaction branch are defined in the same manner. The three descriptor vectors are then concatenated and fed into a lightweight weight-prediction network ϕ(·). A Softmax function is used to obtain the fusion weights for the three feature streams:

(7) $$[w_{r},w_{h},w_{c}]=Softmax\left(\phi \left(\left[g(F_{r}),g(F_{h}),g(F_{c})\right]\right)\right),$$

Finally, the fused feature representation is obtained as

(8) $$F^{f}=w_{r}F^{r}+w_{h}F^{h}+w_{c}F^{c},$$

This design allows the model to dynamically adjust the contribution of each feature source according to the modality reliability of each sample, thereby improving its adaptability to samples at different stages of mold spoilage.

#### 2.3.6. Classification Head and Output Strategy

Mask-constrained global average pooling was applied with mask constraints on the fused feature map to obtain a sample-level feature vector, which is then mapped to mold development through a classification head composed of a fully connected layer, ReLU activation function, Dropout mechanism, and output layer. Considering that the degree of mold severity has both classification and ordinal attributes, this study evaluated two different output paradigms.

The first strategy is standard multiclass classification. Let the number of classes be K; the output dimension is therefore also K. During training, the cross-entropy loss is used:

(9) $$L_{\mathrm{CE}}\;=\;-\sum_{k=1}^{K}y_{k}\log{\hat{y}}_{k},$$

where $y_{k}$ denotes the one-hot encoding of the true class, and ${\hat{y}}_{k}$ denotes the predicted probability of the model.

Another approach is to use the ordinal learning paradigm. This strategy treats the grade label as a set of cumulative binary decisions indicating whether the sample exceeds each grade threshold. The output dimension is K − 1, and the model is trained using a cumulative binary cross-entropy loss. During inference, the final grade is recovered according to the cumulative number of thresholds exceeded. Except for experiments specifically designed to compare training objectives, the standard multiclass classification strategy is used by default in the main experiments of this study. The detailed comparison results are presented in Section 3. Ultimately, the integrated MFGF-Net model achieved fine-grained mold severity grading of individual wheat grains.

### 2.4. Model Training and Evaluation Settings

To reduce data leakage arising from repeated observations of the same physical kernel, all dataset partitions were performed at the kernel level using unique kernel identifiers. The 400 tracked kernels were divided into training, validation, and test sets at a ratio of 70:15:15, corresponding to 280, 60, and 60 kernels, respectively. All 13 temporal observations from a given kernel were assigned exclusively to one subset; therefore, no physical kernel appeared in more than one partition. This split yielded 3640 training pairs, 780 validation pairs, and 780 test pairs. The same kernel-level partitioning strategy was used for all comparative and repeated experiments to ensure strict comparability across models. This strategy prevents direct kernel-level leakage between training and test sets. However, repeated observations of each kernel over 13 consecutive incubation days may still introduce temporal autocorrelation, reflecting the continuous nature of mold progression. To evaluate the potential influence of this dependence on the reported performance, an additional single-observation-per-kernel evaluation was conducted. In each run, one time point was randomly sampled from each test kernel, producing 60 kernel-independent test observations. This temporal subsampling procedure was repeated multiple times, and the mean performance with confidence intervals was reported.

Model training was performed on an NVIDIA GeForce RTX 4090 GPU with 24 GB of memory, using PyTorch (version 2.9.0) as the deep-learning framework. AdamW was used as the optimizer, with an initial learning rate of $2\;\times \;10^{-4}$, a batch size of 8, 50 training epochs, and a weight-decay coefficient of $1\;\times \;10^{-4}$. Gradient clipping with a maximum norm of 5.0 was applied to stabilize the training process. To improve the robustness of the results, all experiments were repeated three times, and the mean and standard deviation of the test-set metrics were reported. No additional data augmentation was used during training. Because MFGF-Net relies on accurate local RGB–HSI registration, strong geometric augmentations, including random cropping, large-angle rotation, affine transformation, and modality-specific independent augmentation, were not adopted. This strategy was used to preserve the registered local spatial correspondence among RGB images, hyperspectral cubes, and foreground masks. The validation and test sets were evaluated using the original preprocessed samples.

Model performance was evaluated using three metrics: accuracy, macro-F1 score, and mean absolute error (MAE). Accuracy was used to measure the overall classification correctness, Macro-F1 was used to assess balanced recognition performance across classes, and MAE was used to quantify the deviation in grade prediction. MAE is defined as follows:

(10) $$\mathrm{MAE}\;=\;\sum_{n=1}^{N}|{\hat{y}}_{n}\;-\;y_{n}|,$$

where N denotes the number of test samples, and ${\hat{y}}_{n}$ and ${\;y}_{n}$ denote the predicted grade and true grade of the *n* sample, respectively. For results involving multiple independent repeated experiments, this study consistently reports the results in the form of mean ± standard deviation over three runs.

## 3. Results

To evaluate the effectiveness of the constructed dataset and MFGF-Net in wheat mold grading, we verified the reliability of the dataset construction and preprocessing workflow, as well as the effectiveness of the foreground mask and background suppression strategies. Next, we assessed the overall performance of MFGF-Net and conducted comparative validation on publicly available RGB–HSI datasets and our self-constructed wheat dataset, comparing the proposed method with traditional machine learning methods, unimodal deep learning models, and multimodal deep fusion models. Finally, through ablation experiments, we explored the impact of different training objectives, modal inputs, fusion strategies, core modules, and sample-level adaptive fusion weights on model performance.

### 3.1. Effectiveness of the Dataset and Preprocessing Workflow

#### 3.1.1. A/B Synchronization and Validity of Proxy Labels

Because the process of wheat mold development is continuously evolving, and reference tests such as fungal spore counts are destructive, it is impossible to perform both non-destructive imaging and ground truth annotation on the same physical grain. Therefore, this study adopts an A/B dynamic parallel design to obtain supervisory signals, transferring the microbiological reference results from Group B to the images of Group A as time-matched surrogate labels. Under this weakly supervised paradigm, verifying the synchronous evolution characteristics of the two groups of grains during the cultivation process is a necessary prerequisite for subsequent model training.

**Figure 5:**
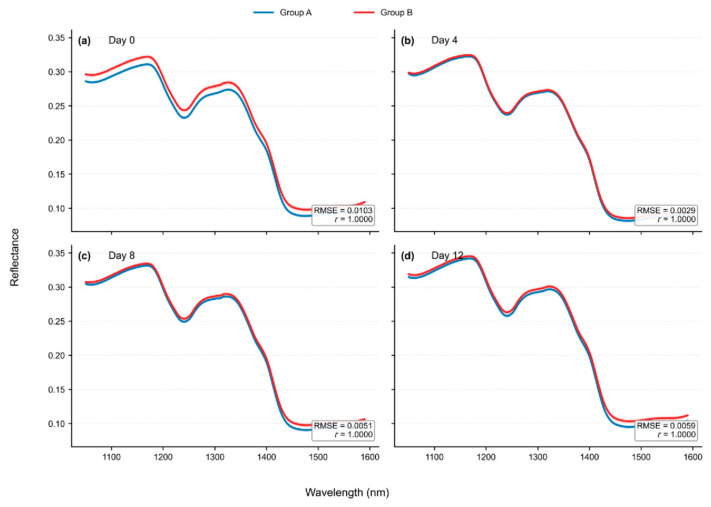
Mean hyperspectral reflectance curves of samples from Groups A and B at different storage time points. The blue and red curves represent Groups A and B, respectively. Subfigures (**a**)–(**d**) correspond to Day 0, Day 4, Day 8, and Day 12, respectively.

We evaluated the intergroup consistency through the average spectral trends. Visualization results at four representative cultivation stages (Days 0, 4, 8, and 12) show that the spectral curves of the paired groups exhibit parallel wavelength variation trends without systematic bias, supporting the use of using Group B’s measurements as weak supervisory references for Group A, as shown in Figure 5. These observations support using the measurements from Group B as weak reference information for the corresponding samples in Group A.

**Figure 6:**
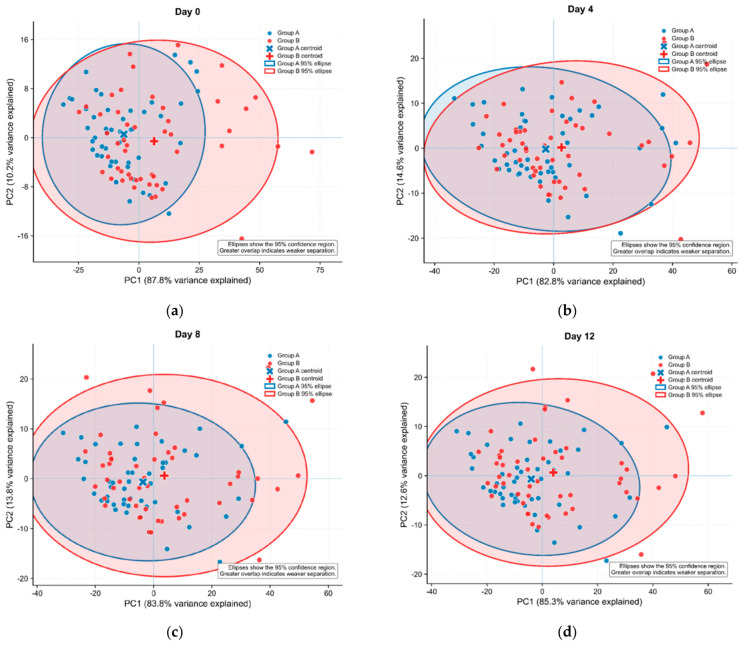
Comparison of PCA score plots for samples from Groups A and B at different incubation time points. The figure shows the PCA score distributions of Groups A and B based on hyperspectral features at four time points: Day 0, Day 4, Day 8, and Day 12. Blue and red points denote samples from Groups A and B, respectively. Cross markers indicate the centroid of each group. (**a**) PCA score plot of samples from Groups A and B on Day 0. (**b**) PCA score plot of samples from Groups A and B on Day 4. (**c**) PCA score plot of samples from Groups A and B on Day 8. (**d**) PCA score plot of samples from Groups A and B on Day 12.

We further examined the consistency between Group A and Group B at the level of low-dimensional distribution structure. As shown in Figure 6, at each time point, samples from both Group A and Group B exhibited substantial overlap in the principal component space, with their centroids closely adjacent to each other and no stable intergroup separation boundary formed. The confidence ellipse of Group B was slightly larger, indicating higher within-group dispersion. However, this difference mainly manifests as an expansion of the distribution range rather than a shift in the overall distribution center. Collectively, the mean spectral analysis and PCA distribution results indicate that Groups A and B followed similar spectral evolution patterns under the same incubation conditions, providing support for subsequent proxy-label transfer.

#### 3.1.2. Quantitative Validation of Proxy Label Uncertainty

To quantitatively evaluate the intrinsic uncertainty of A/B dynamic parallel proxy labels and demonstrate their applicability as risk-level indicators, this study conducted an additional small-scale validation experiment. The 30 replicates were distributed across the four severity stages, and each replicate consisted of one imaged kernel followed by immediate destructive spore-count measurement. Specifically, a portion of individual kernels was selected and subjected to non-destructive imaging for proxy label assignment, followed immediately by destructive testing to determine the ground-truth spore counts.

As shown in Table 1, the overall stage-level agreement of the proxy labeling strategy reached 87.5% ± 4.3% throughout the entire incubation period, and its temporal dynamic pattern was highly synchronized with the actual mold growth progression. In the Safe and Critical stages, the label agreement remained steadily at 91.5–100.0%, and the coefficient of variation (CV) of spore counts ranged from 8.4% to 15.8%, indicating stable microbial growth and low inter-kernel variability at this stage.

As the infection further progressed to the Hazardous and Severe stages, the label agreement decreased to 82.3% and 71.2%, respectively. Meanwhile, the variance among experimental replicates expanded significantly, with the standard deviation of the spore-count CV reached 6.8% in the Severe stage. This variation suggests increased biological heterogeneity among kernels during the later stages of mold development. The research results ultimately verify that although micro-heterogeneity in the late infection stage introduces a certain degree of data noise, the proxy labels still possess a reliable risk-bounding capability.

A clear stage-dependent trend was observed: grade consistency decreased progressively from 100.0% in the Safe stage to 71.2% in the Severe stage, whereas the spore-count coefficient of variation increased from 8.4% to 39.6%, indicating that proxy-label reliability declined as biological heterogeneity increased during mold progression. Proxy-label uncertainty therefore exhibits a distinct stage-dependent distribution throughout the mold development process. This stage-specific uncertainty is a direct manifestation of inherent biological heterogeneity among kernels during late-stage fungal colonization. Under identical incubation conditions and durations, inherent differences in local microenvironmental moisture distribution, initial fungal colonization sites, and final spore loads among individual kernels lead to inevitable class boundary ambiguity between Hazardous and Severe samples. Thus, the time-matched proxy labels used in this study represent population-level risk indicators rather than absolute microbiological ground truth at the individual kernel level. Such label noise leads to softened decision boundaries between adjacent late-stage risk levels and significantly increases the probability of ordinal neighboring misclassifications. Label inconsistency is almost exclusively confined to adjacent high-risk stages, with rare cross-class confusion between Safe and Severe samples. Errors introduced by proxy labels are highly structural rather than random. This study employs accuracy, Macro-F1 score, and mean absolute error (MAE) as multi-dimensional evaluation metrics. MAE specifically quantifies the ordinal deviation of prediction results and effectively evaluates the discrepancy between model outputs and true risk levels, aligning with the inherent ordinal nature of mold grading tasks. The proxy-label strategy based on dynamic parallel A/B design cannot fully replace destructive kernel-level microbiological ground truth annotation. It provides a practical and effective weakly supervised solution for single-kernel mold dynamic monitoring—a field long plagued by label scarcity.

#### 3.1.3. Evaluation of RGB–HSI Registration Quality

Microscopic RGB and hyperspectral modalities exhibit differences in imaging mechanisms, spatial resolutions, and boundary morphologies, which inevitably lead to spatial mismatch issues during direct feature interaction. To reduce cross-modal misalignment and support paired sample construction, this study employs an Enhanced Correlation Coefficient (ECC)-based affine registration algorithm to achieve spatial alignment between modalities.

**Figure 7:**
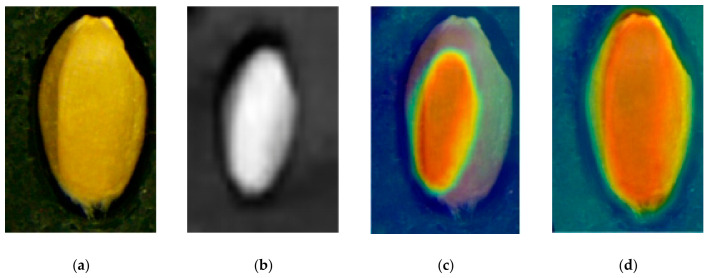
Structural overlay results of RGB and HSI images before and after registration. (**a**) RGB reference image; (**b**) HSI reference image; (**c**) RGB–HSI overlay result before registration; (**d**) RGB–HSI overlay result after registration.

The registration results are shown in Figure 7. After registration, the kernel contour, top region, germ tip, and ventral crease in the RGB images showed good overlap with the corresponding structures in the hyperspectral images. This result indicates that the method effectively reduced the overall geometric displacement caused by differences in imaging viewpoint and scale. Slight local residuals were still observed in a few edge regions, but the main body of each kernel had established a relatively stable spatial correspondence.

To further quantify registration accuracy, we selected three stable reference points—the kernel top, germ tip, and center of the ventral crease—and calculated the Euclidean distance error between corresponding points. As shown in Table 2, the mean Euclidean key-point error after registration was 3.56 pixels. The errors across the different structural points were generally of a similar order of magnitude, with no obvious local mismatch showing abnormally large deviation. Together with the qualitative overlay results, this level of registration accuracy provides an acceptable geometric basis for subsequent local cross-modal interaction.

We further evaluated the registration quality. Representative paired samples were randomly selected from the four mold stages, with 20 samples from each stage. For each selected sample, three non-collinear landmarks were annotated on visually identifiable kernel structures to calculate the post-registration landmark error. The use of three non-collinear landmark pairs follows the geometric requirement of two-dimensional affine transformation estimation and has been adopted in previous image registration studies for estimating affine transformation parameters [27].

Three evaluation metrics were used in this assessment. First, the landmark root mean square error (Landmark RMSE) was used to quantify the average Euclidean distance between corresponding landmark pairs after registration, where a lower value indicates better local geometric alignment. Second, the Dice coefficient was used to measure the spatial overlap consistency between the RGB foreground mask and the transformed HSI foreground mask, with values closer to 1 indicating higher foreground agreement. Third, the intersection-over-union (IoU) was calculated as the ratio of the overlapping foreground region to the union of the two foreground regions, where a higher value indicates better region-level alignment.

As shown in Table 3, the overall Landmark RMSE was 3.59 pixels, while the Dice coefficient and IoU were 0.928 and 0.866, respectively. Considering that the paired RGB and HSI samples were normalized to a unified spatial size of 384 × 256 pixels, the residual landmark-level deviation remained within a small pixel-level range. The slightly increased Landmark RMSE and reduced Dice/IoU values in the Hazardous and Severe stages indicate that advanced mold development introduced stronger boundary irregularity and foreground-mask uncertainty. Nevertheless, the foreground-region overlap remained acceptable across all mold stages, suggesting that the RGB–HSI registration quality was sufficient to support subsequent foreground-constrained local cross-modal fusion.

#### 3.1.4. Effectiveness of the Foreground Mask and Background Suppression

The original images of wheat kernels inevitably capture irrelevant artifacts, such as edge shadows, local specular reflections, and background textures. Unconstrained deep networks are at risk of confusing these background variations with genuine discriminative features, thereby diverting attention from phenotype changes induced by mold. This study conducted controlled variable ablation experiments to isolate the effect of the proposed spatial constraints, and the results are shown in Table 4.

In the “No mask” setting, the foreground mask was completely removed from all subsequent processing and feature-extraction modules. In contrast, the “No background suppression” setting retained the mask-guided operations in the network while preserving the original image background during input preprocessing. The results show that the complete model achieved the best performance across all three metrics: Accuracy, Macro-F1, and MAE. After removing the foreground mask, Accuracy decreased from 0.9689 to 0.8445, Macro-F1 decreased from 0.9698 to 0.8281, and MAE increased from 0.0593 to 0.5480. These changes indicate that the foreground mask plays an important role in constraining the effective regions. Removing background suppression also degraded model performance, suggesting that background purification helps reduce irrelevant texture and illumination interference in the visual branch.

### 3.2. Model Performance and Comparative Evaluation

After examining the dataset construction and preprocessing workflow, we conducted a comprehensive performance evaluation of MFGF-Net on both public benchmark datasets and the self-constructed wheat dataset. To verify the auxiliary evaluation of the cross-modal fusion module of the proposed local cross-modal fusion strategy, a fusion-reconstruction variant of the network was first constructed and compared with mainstream fusion algorithms on the CAVE and Harvard hyperspectral datasets. Subsequently, the complete MFGF-Net model is further applied to the self-built wheat dataset. From the perspective of fine-grained mold severity grading performance, rigorous comparative experiments are carried out with traditional machine learning algorithms, unimodal deep networks and other multimodal fusion baseline models.

#### 3.2.1. Overall Performance of MFGF-Net

Since the public CAVE and Harvard datasets do not contain wheat mold grade labels, this study only uses the two benchmark datasets to verify the fusion capability of the reconstructed MFGF-Net variant. The evaluation metrics include PSNR, SSIM, SAM and ERGAS. On the CAVE dataset, the model achieves PSNR = 45.40 ± 3.85, SAM = 3.58 ± 0.61, ERGAS = 3.02 ± 0.82 and SSIM = 0.974 ± 0.007. On the Harvard dataset, the results are PSNR = 43.80 ± 3.38, SAM = 3.85 ± 0.62, ERGAS = 3.10 ± 0.82 and SSIM = 0.966 ± 0.010, all demonstrating robust spatial-spectral reconstruction performance. These metrics suggest that the local cross-modal fusion module has basic RGB–HSI representation capability, providing auxiliary support for its subsequent application to mold severity grading.

On the self-built wheat dataset, the complete MFGF-Net demonstrates outstanding performance in mold contamination risk grading. The results are shown in Table 5. The model achieves an accuracy of 0.9689 ± 0.0225, a Macro-F1 score of 0.9698 ± 0.0221, and a mean absolute error of merely 0.0593 ± 0.0429 on the independent test set. The high classification indicators are consistent with the extremely small ordinal prediction error, which verifies that the model can accurately distinguish mold risk levels with continuous gradients.

**Figure 8:**
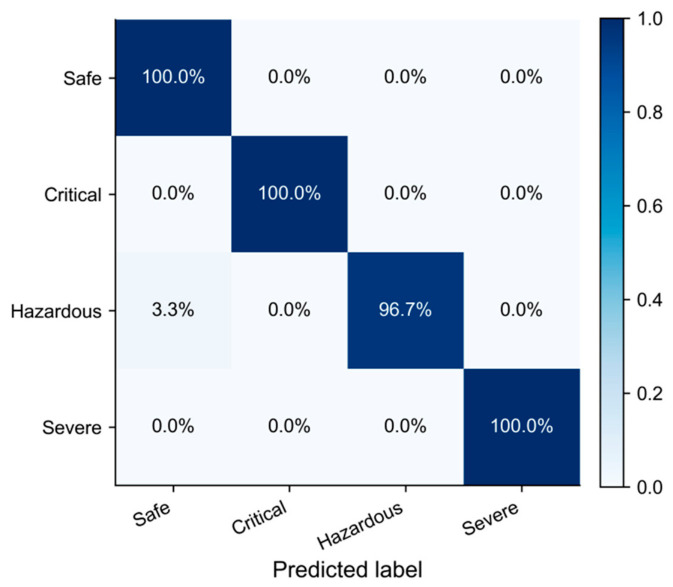
Confusion matrix of MFGF-Net on the test set. The figure shows the classification results for the four mold severity grades—Safe, Critical, Hazardous, and Severe—on the test set. The vertical axis denotes the true labels, the horizontal axis denotes the predicted labels, and the values indicate the proportion of samples from each class predicted as the corresponding class.

To further analyze the model’s recognition ability across different grades, we present the confusion matrix and class-wise evaluation results. As shown in Figure 8, the confusion matrix exhibits a pronounced diagonal pattern, indicating that the model has strong discriminative ability for each mold-spoilage grade. The class-wise results in Table 6 show that the Precision, Recall, and F1-score for the Critical and Severe classes all reached 1.0000. The Safe and Hazardous classes also maintained high F1-scores, suggesting that the model achieved balanced performance across classes. Misclassifications only occurred in a small fraction of samples, mainly distributed in categories with relatively lower class-level performance. Such errors generally appear when mold-induced visual features and spectral signals are faint, ambiguous, or mismatched between different modalities.

To assess whether repeated temporal observations affected the reported performance, we further examined temporal dependence within the test kernels. Although the dataset was split strictly at the kernel level, each test kernel still contributed 13 observations collected over consecutive incubation days. Because mold development is a gradual biological process, these observations cannot be regarded as fully independent, as shown in Table 7.

The fused features of adjacent-day observations showed a high mean cosine similarity of 0.9148 ± 0.0387 across 720 adjacent-day pairs, indicating clear temporal continuity within the same kernel. This result confirms that the original test set should be interpreted as kernel-separated time-point observations rather than as fully independent biological replicates.

A conservative single-observation-per-kernel validation was therefore conducted. In each run, only one time point was randomly selected from each test kernel, and the procedure was repeated 100 times. Under this setting, MFGF-Net achieved an accuracy of 0.9656 ± 0.0224, a Macro-F1 score of 0.9642 ± 0.0241, and an MAE of 0.0668 ± 0.0305. Compared with the original time-point evaluation, performance decreased only slightly, whereas variability increased due to the reduced number of test observations in each run. These results suggest that the high classification performance was not solely attributable to repeated temporal observations from the same kernels.

#### 3.2.2. Auxiliary Evaluation on Public RGB–HSI Datasets

Since there is no standardized paired RGB-hyperspectral dataset for wheat mold severity assessment to date, we conducted auxiliary validation on four public benchmark datasets, namely CAVE, Harvard, ARAD_1K and Agro-HSR, to evaluate the general cross-modal representation capability of the MFGF-Net framework. CAVE and Harvard are commonly used hyperspectral benchmark datasets, ARAD_1K represents a larger general natural-scene RGB–HSI benchmark [28], and Agro-HSR provides an agriculturally relevant reference with more complex textures, irregular morphology, and stronger spectral variations [29]. A fusion-reconstruction variant of MFGF-Net was constructed for this validation. As shown in Table 8, dedicated reconstruction models such as HSRnet and Fusformer achieved better global reconstruction metrics on CAVE and Harvard, as they are specifically optimized for whole-image spatial reconstruction and spectral consistency [30]. Although the MFGF-Net variant showed slightly lower reconstruction performance, it remained competitive and achieved stable results on ARAD_1K and Agro-HSR, with PSNR values of 38.60 ± 1.85 and 37.40 ± 2.05 and SSIM values of 0.965 ± 0.012 and 0.948 ± 0.018, respectively. The lower performance on Agro-HSR may be related to the higher complexity of agricultural samples. Overall, the results demonstrate that MFGF-Net possesses favorable fundamental capability for RGB-hyperspectral cross-modal representation. It should be emphasized that the core strength of MFGF-Net lies in local discriminative fusion for fine-grained mold grading. However, these public datasets do not contain wheat mold grading labels; therefore, the corresponding results can only serve as indirect evidence of the model’s cross-modal representation capability.

#### 3.2.3. Model Comparison on the Self-Constructed Dataset

To verify the advantages of the proposed model, this study conducted a comparative evaluation of MFGF-Net with various models. Traditional machine learning algorithms include Support Vector Machine (SVM) and Partial Least Squares Discriminant Analysis (PLS-DA) based on hyperspectral mean spectra, as well as Random Forest (RF) based on concatenated RGB–HSI features. Unimodal deep learning baseline models consist of ResNet18 based on RGB images, a one-dimensional Convolutional Neural Network (1D-CNN) based on spectra, and HybridSN and 1D-SSFTT networks based on HSI cubes. A unified data partitioning strategy is adopted for all comparative experiments.

As shown in Table 9, traditional machine learning algorithms exhibit inferior overall performance compared with unimodal deep networks, indicating that relying merely on mean spectra or shallow concatenated features cannot effectively characterize the local heterogeneity of mold samples. Among the deep baseline models, hyperspectral-driven models substantially outperform ResNet18 using only RGB data, demonstrating that hyperspectral data is the dominant feature modality in mold discrimination tasks. Although HybridSN and 1D-SSFTT achieve competitive performance, the proposed MFGF-Net still obtains the optimal results across all prediction indicators. The results reveal that independent RGB visual features have limited discriminative capability, and the local cross-modal fusion of RGB and hyperspectral features can effectively resolve the ambiguous boundaries in mold severity grading.

#### 3.2.4. Comparison with Multimodal Fusion Models

This study conducts comparative experiments between MFGF-Net and mainstream multimodal network structures, including feature concatenation + multi-layer perceptron, global cross-attention, and Transformer-based fusion models. As shown in Table 10, the basic feature concatenation scheme (Concat + MLP) performs far worse than various attention-based fusion methods, and it may be insufficient to model fine-grained cross-modal interactions between RGB and hyperspectral data. Although global cross-attention and Transformer models improve classification indicators through explicit cross-modal interaction, their final performance is still inferior to MFGF-Net. This performance gap indicates that global fusion methods have inherent shortcomings and are vulnerable to interference from residual background noise and spatial misalignment. MFGF-Net constrains cross-modal attention to the local neighborhood of the foreground, which better conforms to the developmental law of wheat mold with subtle features and spatial heterogeneity, thus achieving the optimal mold grading effect.

**Figure 9:**
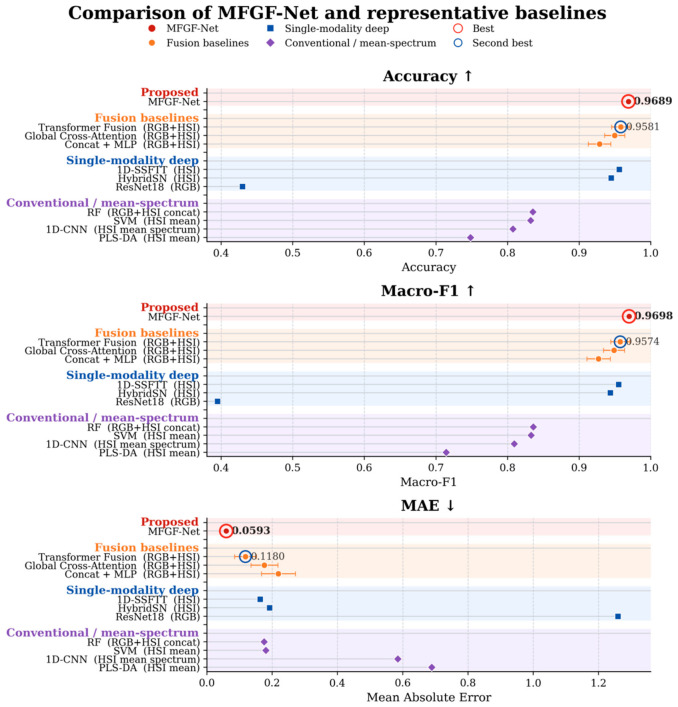
Performance comparison between MFGF-Net and representative baseline methods. The figure shows accuracy, macro-F1 score, and MAE, providing a comprehensive comparison of MFGF-Net with traditional methods, single-modality methods, and fusion baselines.

As shown in Table 11, the paired *t*-tests were conducted based on three independent repeated runs, corresponding to three paired observations (n=3, df=2). The significance level was set to α=0.05, and the reported *p*-values were used as supplementary statistical evidence. MFGF-Net outperforms Transformer Fusion across all three evaluation metrics: classification accuracy, Macro-F1 score, and MAE. This result indicates that constraining cross-modal attention within the local neighborhood of the foreground effectively mitigates interference from background clutter and misalignment errors, thereby significantly improving the fine-grained grading performance of wheat mold. To provide an overall visual comparison across all representative baselines, including conventional mean-spectrum models, single-modality deep models, fusion baselines, and the proposed MFGF-Net, the performance results in terms of accuracy, Macro-F1, and MAE are summarized in Figure 9.

### 3.3. Ablation Experiments

#### 3.3.1. Performance Comparison of Different Training Objectives

Wheat mold severity grades have both discrete categorical attributes and ordinal relationships. To compare the applicability of different training objectives in this task, this paper conducts a comparative evaluation of standard cross-entropy loss and the ordinal learning objective, and the results are shown in Table 12.

The experimental results show that cross-entropy loss (CE) is superior to the ordinal learning objective (Ordinal) in all three metrics. Specifically, the accuracy of cross-entropy loss is 0.9689, the Macro-F1 is 0.9698, and the mean absolute error is 0.0593. In contrast, the corresponding Accuracy, Macro-F1, and mean absolute error of the ordinal learning objective are 0.9185, 0.8234, and 0.2440, respectively. The results indicate that under current data conditions, directly learning the discriminative boundaries between discrete categories is more suitable for this research task than explicitly modeling the ordinal relationships among grades.

Wheat mold progression is intrinsically ordinal. However, the weaker performance of the ordinal objective may be related to the weakly supervised nature of the labels and the non-uniform separability among stages. The proxy labels used in this study represent time-matched stage-level risk categories rather than directly measured kernel-level continuous severity. Thus, enforcing cumulative ordinal thresholds may amplify sensitivity to label uncertainty, particularly near adjacent stage boundaries. Moreover, visual and spectral changes during mold development are not necessarily evenly spaced: early-stage alterations are subtle, whereas later-stage fungal growth may produce more abrupt phenotypic and biochemical shifts. Under these conditions, cross-entropy can more flexibly learn discrete stage-specific decision boundaries, making it more suitable for the weakly supervised fine-grained classification setting of this study.

#### 3.3.2. Ablation of Different Modalities and Fusion Strategies

To evaluate the respective contributions of single modalities and different fusion paradigms, this study evaluated four ablation settings: RGB-only, HSI-only, simple fusion, and late fusion. As shown in Table 13, the model relying solely on the hyperspectral modality substantially outperforms the RGB-only model, further verifying that hyperspectral features act as the dominant discriminative modality in mold severity grading tasks.

Both simple fusion and late fusion baseline models present suboptimal performance, indicating that basic feature concatenation or decision-level averaging fails to fully explore cross-modal synergistic correlations. The complete MFGF-Net achieves the optimal results across all evaluation metrics, demonstrating that the superior performance gain originates directly from the localized interactive fusion mechanism of complementary spatial-spectral features.

#### 3.3.3. Ablation of Core Modules

To quantify the contribution of each component to the model structure, structural ablation experiments were carried out on the MDCLA module, FASR module, and sample-level adaptive fusion head of MFGF-Net. The results are presented in Table 14.

After removing the MDCLA module, the model performance decreased substantially, indicating that local cross-modal interaction is the core architecture of the network. This module performs local attention-based interaction between RGB phenotypic features and HSI spectral-spatial features under foreground-mask constraints, which fundamentally improves the ability to distinguish subtle morphological differences induced by mold infection.

When the sample-level adaptive fusion module was removed, the overall accuracy and Macro-F1 value of the “No Gating” method were lower than those of the complete model, and the increase in MAE was more obvious. This result shows that the sample-level gating mechanism can dynamically adjust the contributions of RGB features, HSI features, and cross-modal interactive features according to the modal reliability of different samples, thereby reducing ordinal prediction errors in mold severity grading.

After removing the FASR module, the accuracy and Macro-F1 value decreased to 0.9320 ± 0.0364 and 0.9290 ± 0.0107, respectively. These results indicate that the foreground-aware spectral recalibration technology effectively suppresses the interference from background regions and redundant bands in the process of hyperspectral feature extraction, allowing the model to focus more accurately on valid spectral response features associated with mold spoilage.

**Figure 10:**
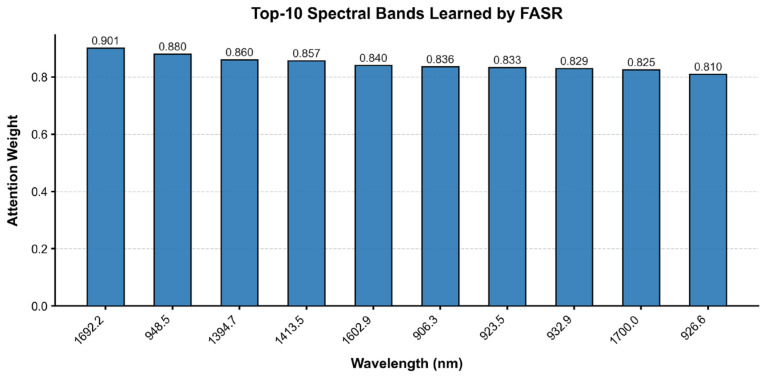
Top 10 spectral bands with the highest channel-wise attention weights learned by FASR. The weights reflect the model’s focus on informative spectral signatures associated with intrinsic biochemical changes underlying wheat mold growth.

To examine whether the spectral recalibration learned by the FASR was physiologically meaningful, the top 10 spectral bands were ranked according to the average channel-wise attention weights across the test set (Figure 10). All selected bands showed attention weights greater than 0.810, indicating that the spectral contribution was concentrated within a limited set of wavelengths rather than being uniformly distributed across the hyperspectral range. The strongest responses occurred at 1692.2 nm, 948.5 nm, and within the adjacent 1394.7–1413.5 nm region. These bands are mainly associated with the first overtone of C–H stretching, the third overtone of O–H/C–H stretching, and the first overtone of O–H stretching, respectively [31,32,33]. This attention pattern is broadly consistent with the expected biochemical trajectory of wheat kernel deterioration during fungal colonization. The O–H-related response around 1400 nm may reflect changes in water binding, migration, and local redistribution within the kernel, which are closely related to fungal activity under high-humidity storage conditions. The C–H-related response near 1692.2 nm may be associated with alterations in carbon-rich storage reserves, including starch, lipids, and other organic substrates that are progressively utilized or modified during mold development. Protein- and cell-wall-related changes may also contribute to the overall near-infrared response, although the broad and overlapping nature of NIR absorption prevents these bands from being assigned to a single biochemical constituent. These spectral responses also characterize the biochemical background associated with mycotoxin risk development, as fungal secondary metabolism is closely influenced by water availability, substrate utilization, colonization intensity, and stress-related physiological conditions. Accordingly, the wavelengths selected in this study can be interpreted as spectral indicators of mold-induced biochemical perturbations and storage-risk evolution. Taken together, the FASR attention profile suggests that the model preferentially emphasizes spectral regions with plausible biological relevance to moisture redistribution, substrate degradation, and progressive fungal colonization.

#### 3.3.4. Analysis of Sample-Level Adaptive Fusion Weights

To analyze the role of the sample-level gating mechanism in final discrimination, this study quantified the adaptive weight distribution of unimodal (RGB, HSI) branches and cross-modal interaction branches on the test set, and the results are presented in Table 15.

**Figure 11:**
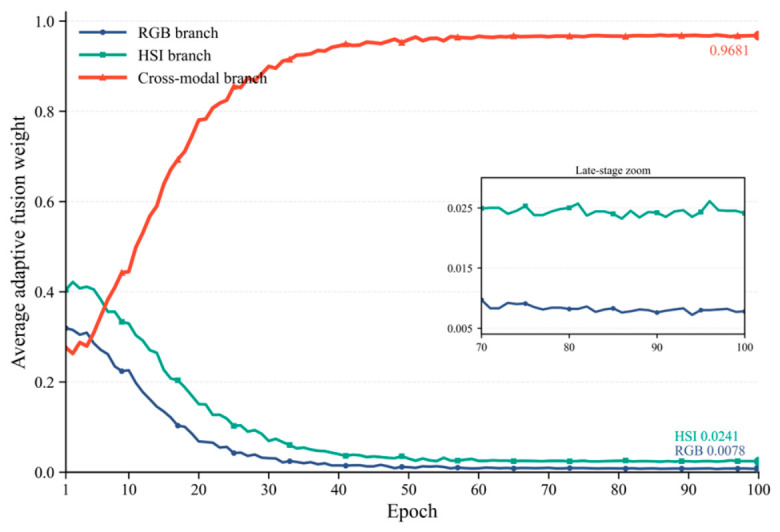
Evolution of sample-level adaptive fusion weights during training. The average adaptive fusion weights assigned to the RGB, HSI, and cross-modal branches were recorded over 100 training epochs. The HSI branch exhibited a relatively larger contribution at the initial optimization stage, consistent with the strong discriminative role of hyperspectral responses. As training progressed, the model increasingly relied on the local RGB–HSI interaction branch, suggesting that the gated fusion module progressively learned to exploit registered cross-modal correspondences. In the late training stage, the cross-modal weight stabilized at approximately 0.968, while the RGB and HSI branches retained small but non-zero auxiliary contributions.

In the overall test set, the cross-modal interaction branch dominates the gating weight distribution with a peak average weight of 0.9682, which is far higher than those of the independent RGB and HSI unimodal branches. For correctly classified samples, the cross-modal weight reached 0.9723, whereas for misclassified samples, it decreased to 0.8787, accompanied by a compensatory increase in unimodal weights. To further illustrate the dynamic behavior of the gating mechanism, Figure 11 presents the evolution of the average fusion weights over training epochs. In the late training stage, the cross-modal branch gradually became dominant and stabilized at approximately 0.968, while the RGB and HSI branches maintained relatively small but non-zero auxiliary contributions. This indicates that the model progressively learned to rely on local RGB–HSI association features while preserving complementary information from individual modalities.

The adaptive fusion weights are clearly biased toward the cross-modal branch, indicating that fine-grained mold grading mainly relies on local RGB–HSI joint representations rather than on RGB or HSI features alone. The cross-modal branch encodes local correspondences between visible phenotypic cues and hyperspectral responses, which explains its high fusion weight. The highly concentrated weights suggest a tendency of the gating mechanism to favor the cross-modal branch, but this does not necessarily mean a complete collapse. The RGB and HSI branches still participate in constructing the cross-modal representation and may provide auxiliary information when visual symptoms and spectral responses are not synchronized. The limited contribution of unimodal output branches also suggests that the model could be further simplified, for example, by retaining the local RGB–HSI interaction backbone while reducing dependence on low-contribution unimodal output streams.

## 4. Discussion

This study constructs a fine-grained discrimination framework based on single-kernel RGB–HSI paired data and weakly supervised labels. Wheat mold spoilage is characterized by continuous gradual variation and spatially localized diffusion, accompanied by significant heterogeneity among individual kernels. Accordingly, it can be conceptually regarded as an ordinal risk-grading problem, although the current proxy-label setting favored a categorical cross-entropy objective. That considers infection stages. Experimental results confirm that the synergistic effect of A/B parallel proxy labels, foreground-constrained multimodal registration, and local cross-modal interaction provides core support for the superior grading performance of the proposed model.

### 4.1. Reliability of Weakly Supervised Labels

There is an inherent contradiction between continuous nondestructive imaging detection and destructive microbiological reference testing, which makes it impossible to conduct simultaneous ground truth labeling on the same batch of single-kernel samples. To this end, this study adopts an A/B dynamic parallel experimental design and transfers the fungal spore count grades of destructive Group B samples to time-matched imaging samples in Group A. This paradigm is essentially a weakly supervised labeling strategy based on parallel group references [34]. The provided indirect support for the proxy-label transfer of this label transfer mechanism. The average spectral curves and PCA distributions of the two groups present consistent temporal variation trends with highly overlapped spatial features, and there is no systematic offset between groups.

The overall grade consistency of proxy labels is 87.5% ± 4.3%, while the MFGF-Net model still achieves a high classification accuracy of 0.9689. In the weakly supervised learning scenario, the discrepancy between the two reflects the excellent representation learning capability of the model without methodological contradiction. Although biological heterogeneity brings label uncertainty, the proposed multimodal model can learn the consistent variation law of wheat mold spoilage by introducing masked foreground constraints and the MDCLA local cross-modal interaction mechanism, and effectively avoid overfitting to local noisy supervision signals. The decrease in proxy label consistency under Hazardous and Severe stages is closely related to the significant increase in the variability of spore quantity, indicating that fungal colonization within individual kernels is increasingly heterogeneous. Therefore, the observed uncertainty should be regarded as an inherent characteristic of the natural mold progression rather than a defect of the proxy labeling strategy. The research results further indicate that the proposed weakly supervised multimodal framework can learn robust RGB–HSI feature representations in the presence of moderate label ambiguity.

### 4.2. Cross-Modal Alignment and Dataset Validation

RGB images can acquire apparent phenotypic characteristics such as color, texture, and morphology of wheat grains, while hyperspectral data contain continuous internal biochemical information [35,36]. Nevertheless, there exist inherent differences in spatial resolution and imaging mechanism between the two. Without grain-level matching and size normalization, spatial misalignment will weaken the effect of local feature interaction.

Accurate spatial region selection is a key prerequisite for guaranteeing the recognition stability of grain-level spectral imaging [37,38]. In this study, fixed array arrangement, grain number sequence matching, and ECC affine registration are adopted to achieve precise spatial alignment of samples, which lays a solid geometric foundation for cross-modal feature fusion. Meanwhile, foreground masking and background suppression are indispensable. Redundant background textures and edge shadows may interfere with model discrimination [24], while foreground masking provides a unified spatial constraint for spectral statistics, cross-modal local interaction, and sample pooling. Therefore, the excellent performance of MFGF-Net is the result of the synergy of accurate spatial matching and alignment, effective region constraint, and network architecture.

Public benchmark datasets provided auxiliary evidence that the fusion module has basic RGB–HSI reconstruction capability. The CAVE and Harvard datasets are oriented to the global reconstruction of the whole image, whereas grain mold discrimination is more dependent on subtle local lesion regions in the foreground. The mask constraint and local cross-modal fusion mechanism can guide the network to focus on lesion areas. Although the adapted reconstruction variant did not achieve the best global reconstruction metrics, the classification and ablation results on the self-constructed wheat dataset indicate that the local fusion design is beneficial for fine-grained mold severity grading. Experiments on the self-developed dataset show that MFGF-Net is comprehensively superior to traditional algorithms, unimodal networks, and global fusion baseline models. Therefore, these results should be interpreted within the scope of general RGB–HSI fusion feasibility, and further validation on independent wheat mold grading datasets is still required.

### 4.3. Discriminative Contribution of Local Cross-Modal Fusion

Comparative experiments showed that HSI features served as the dominant discriminative modality, likely because spectral responses are associated with biochemical changes during wheat mold development. Nevertheless, the performance of the MFGF-Net fusion model suggests that under the strict constraints of spatial registration and foreground masking, local RGB features can provide key complementary cues and effectively correct the ambiguous decision boundaries inherent to single hyperspectral classification [23].

Different from the global attention mechanism and basic feature concatenation fusion methods, the structural superiority of MFGF-Net stems from its spatial constraint design. The local cross-modal attention mechanism avoids the interference of residual background noise and contour misalignment on the fusion process, and establishes strict cross-modal feature correspondence only within the foreground region. This local focusing characteristic is consistent with the biological attributes of mold occurrence: the transition boundaries between mold grades are ambiguous, lesion variations are on a microscopic scale, and the spatial deterioration of grain mold exhibits strong heterogeneity.

Ablation experiment results indicate that the performance improvement of the model does not derive from simple modality stacking. The isolated removal of MDCLA, FASR, and adaptive gating modules leads to a precipitous drop in model accuracy, which demonstrates profound interdependence among all modules. The synergistic coupling of foreground-aware spectral filtering, local cross-modal anchoring and dynamic reliability gating enables the model framework to decode the subtle multi-dimensional characteristics of grain mold spoilage.

Further analysis of the misclassified samples in the results indicates that such errors are not solely due to defects in the model itself, but are caused by the combined effect of decreased modal reliability and the inherent uncertainty of weakly supervised labels. From the perspective of modalities, some wheat kernels exhibit subtle phenotypic variations in RGB images or show unstable spectral responses in hyperspectral data, resulting in insufficient local complementary features between the RGB and hyperspectral modalities, making accurate classification difficult. From the perspective of labels, the mold grading labels are calibrated based on temporally matched microbial reference data, without conducting one-to-one destructive testing for each imaged kernel. The natural biological heterogeneity among individual kernels inevitably introduces proxy label noise, a phenomenon that is particularly evident in samples located near the boundaries of adjacent mold grades.

### 4.4. Limitations and Future Applications

The proposed MFGF-Net achieves favorable detection performance under established experimental conditions, while this study still has several limitations.

1. All samples used in this study were collected from a single batch of wheat under controlled temperature and humidity environments. Such a data acquisition strategy ensures the temporal consistency of RGB images, hyperspectral images, and microbial reference measurements, yet it restricts the diversity of biological and environmental features covered in the training dataset. In practical grain storage scenarios, wheat mold development is affected by various factors, including wheat cultivar, harvest year, storage conditions, and fungal community composition. Variations in these factors can alter the visual appearances and spectral responses of mold-infected wheat kernels, potentially leading to domain shifts when the model is deployed in unseen real-world scenarios.

2. The weakly supervised labeling strategy is used to resolve the conflict between continuous non-destructive imaging and destructive microbiological testing. The A/B parallel experimental design transfers time-matched microbiological reference results to continuously imaged kernels, making it possible to construct a single-kernel RGB–HSI dynamic mold dataset. This method is suitable for mold monitoring tasks in which direct kernel-level annotation is difficult to obtain. Its limitation is that, in adjacent mold severity levels, inter-kernel heterogeneity weakens the correspondence among incubation duration, microbiological grading results, and single-kernel deterioration. Future studies may introduce confidence-aware learning, soft-label supervision, uncertainty estimation, and independent dataset validation to further reduce the influence of proxy-label noise on model training and evaluation.

3. The sample-level adaptive fusion module can integrate RGB, HSI, and cross-modal features according to the modal reliability of different samples. The fusion weights show that the cross-modal branch contributes most to the final discrimination, indicating that local RGB–HSI interaction is the main source of discriminative information for fine-grained mold grading. The RGB and HSI unimodal branches still play auxiliary roles, especially when visual symptoms and spectral responses are not synchronized, thereby enhancing prediction stability. The current fusion weights are relatively concentrated, indicating that the gating mechanism still has room for improvement in utilizing complementary information from multiple branches. Future work may introduce weight-balance regularization or confidence-guided gating to avoid excessive concentration of fusion weights.

4. The RGB–HSI fusion framework can be used for non-destructive fine-grained mold grading. Compared with RGB-only detection, paired hyperspectral imaging provides richer spectral information, but also brings larger data volume, higher wavelength calibration requirements, and more complex preprocessing procedures, including single-kernel localization, foreground segmentation, and cross-modal registration. These steps are relatively stable under laboratory conditions, but in industrial sorting environments they are easily affected by kernel movement, posture variation, illumination fluctuation, imaging speed, and hardware cost. Future applications may focus on key wavelength selection, compact multispectral systems, fast registration algorithms, and online detection workflows.

5. Future work will expand the dataset by incorporating wheat samples of different batches, cultivars, harvest years, and storage regions, as well as naturally contaminated grain samples acquired by diverse imaging devices, to comprehensively validate model generalization. Key wavelength selection, informative spectral feature migration for compact multispectral systems, model lightweighting, accelerated registration algorithms, and online workflow optimization can effectively improve imaging efficiency and industrial practicability [39,40,41]. Beyond engineering deployment, integrating prior knowledge of storage conditions and fungal growth mechanisms with causal explanation methods may further improve the biological plausibility, interpretability, and cross-scenario transferability of model predictions [42,43].

## 5. Conclusions

This study proposes a weakly supervised local cross-modal discrimination framework based on paired single-kernel RGB–HSI data for non-destructive single-kernel mold-risk grading in wheat. Because continuous non-destructive imaging and destructive microbiological testing cannot be repeatedly performed on the same physical kernel, an A/B dynamic parallel experimental design was established. Fungal spore-count grades from the destructive reference group were transferred to time-matched imaging samples as weak proxy labels for modeling progressive mold severity. A standardized preprocessing pipeline, including single-kernel cropping, foreground segmentation, RGB–HSI registration, background suppression, and data packaging, was further developed to improve multimodal spatial correspondence and foreground-region constraints.

Based on this dataset, MFGF-Net was developed to integrate RGB phenotypic cues and HSI spectral responses through foreground-aware spectral recalibration, mask-guided local cross-modal interaction, and sample-level adaptive fusion. Spectral analyses of the A/B groups showed broadly consistent temporal evolution between the parallel groups, supporting the use of time-matched proxy labels. On the self-constructed wheat RGB–HSI dataset, MFGF-Net achieved an accuracy of 0.9689 ± 0.0225, a Macro-F1 score of 0.9698 ± 0.0221, and an MAE of 0.0593 ± 0.0429, outperforming traditional machine-learning methods, unimodal deep models, and representative multimodal fusion baselines. Ablation experiments further indicated that the performance improvement was attributed to the combined effects of foreground constraints, spectral recalibration, local cross-modal fusion, and adaptive feature weighting.

Several limitations should be acknowledged in this study. The dataset was collected from a single experimental batch under controlled laboratory incubation conditions, which may not fully represent the biological, cultivar, geographic, and environmental variability of practical granary storage. In addition, the proxy labels were derived from time-matched destructive microbiological references rather than direct kernel-level measurements, introducing label uncertainty, particularly in advanced mold stages with stronger inter-kernel heterogeneity. External validation on independent large-scale datasets from diverse storage environments is still required. From a practical perspective, the current RGB–HSI workflow requires paired imaging hardware, stable illumination, wavelength calibration, single-kernel localization, foreground segmentation, and RGB–HSI registration; therefore, the proposed framework is currently more suitable for laboratory-based application, while future industrial deployment will require compact spectral imaging systems, faster preprocessing workflows, and lightweight models.

Future work should focus on expanding the dataset to cover different wheat varieties, harvest seasons, storage conditions, and imaging devices; incorporating uncertainty-aware learning or soft-label learning to reduce weak-label noise; selecting key spectral bands for compact multispectral implementation; and improving model lightweighting and online deployment capabilities for practical grain storage monitoring.

## Figures and Tables

**Table 1:** Grade consistency and spore count coefficient of variation of proxy labels across different mold spoilage stages.

Mold Spoilage Stage	Time Range (Days)	Grade Consistency (%)	Spore Count CV Value (%)
Safe	0–3	100.0 ± 0.0	8.4 ± 1.2
Critical	4–7	91.5 ± 5.8	15.8 ± 2.7
Hazardous	8–10	82.3 ± 8.4	27.9 ± 4.3
Severe	11–12	71.2 ± 10.5	39.6 ± 6.8
Overall Average	0–12	87.5 ± 4.3	22.9 ± 4.1

**Table 2:** Registration-error statistics for cross-modal key structural points.

Feature Point	RGB Reference Coordinate	HSI Reference Coordinate	Euclidean Distance Error
Point 1	(179, 25)	(176, 22)	4.24
Point 2	(175, 248)	(177, 250)	2.83
Point 3	(104, 103)	(106, 100)	3.61
Mean Euclidean error			3.56

**Table 3:** Lightweight evaluation of RGB–HSI registration quality across mold stages.

Mold Stage	Number of Samples	Landmark RMSE/Pixel	Dice	IoU
Safe	20	3.12 ± 0.64	0.943 ± 0.021	0.892 ± 0.037
Critical	20	3.38 ± 0.71	0.936 ± 0.024	0.880 ± 0.041
Hazardous	20	3.61 ± 0.82	0.925 ± 0.030	0.861 ± 0.049
Severe	20	4.23 ± 0.96	0.907 ± 0.039	0.831 ± 0.061
Overall	80	3.59 ± 0.88	0.928 ± 0.032	0.866 ± 0.052

**Table 4:** Effectiveness comparison of foreground-mask and background-suppression strategies.

Method	Accuracy	Macro-F1	MAE
Full	0.9689 ± 0.0225	0.9698 ± 0.0221	0.0593 ± 0.0429
No mask	0.8445 ± 0.0308	0.8281 ± 0.0365	0.5480 ± 0.1260
No background suppression	0.9282 ± 0.0221	0.9286 ± 0.0224	0.4260 ± 0.0880

**Table 5:** Overall recognition performance of MFGF-Net on the test set.

Experiment	Accuracy	Macro-F1	MAE
Full model (mean ± std)	0.9689 ± 0.0225	0.9698 ± 0.0221	0.0593 ± 0.0429
Best run	0.9926	0.9917	0.0150

**Table 6:** Class-wise Precision, Recall, and F1-score of MFGF-Net on the test set (one representative run).

Class	Precision	Recall	F1-Score
Safe	0.9677	1.0000	0.9836
Critical	1.0000	1.0000	1.0000
Hazardous	1.0000	0.9667	0.9831
Severe	1.0000	1.0000	1.0000

**Table 7:** Conservative validation under temporal subsampling and adjacent-day feature similarity.

Evaluation Setting	Test Observations	Accuracy	Macro-F1	MAE	Feature Similarity
Original time-point evaluation	780	0.9689 ± 0.0225	0.9698 ± 0.0221	0.0593 ± 0.0429	
Single-observation-per-kernel validation	60 × 100 repeats	0.9656 ± 0.0224	0.9642 ± 0.0241	0.0668 ± 0.0305	
Adjacent-day feature similarity	720 adjacent pairs				0.9148 ± 0.0387

**Table 8:** Auxiliary validation results on public and agricultural RGB–HSI datasets.

Dataset	Method	PSNR	SAM	ERGAS	SSIM
CAVE	HSRnet	50.38 ± 3.38	2.23 ± 0.66	1.20 ± 0.75	0.996 ± 0.0014
CAVE	Fusformer	49.98 ± 8.10	2.20 ± 0.85	2.50 ± 5.21	0.994 ± 0.0111
CAVE	Fusion-reconstruction variant of MFGF-Net	45.40 ± 3.85	3.58 ± 0.61	3.02 ± 0.82	0.974 ± 0.0072
Harvard	HSRnet	48.29 ± 3.03	2.26 ± 0.56	1.87 ± 0.81	0.988 ± 0.0064
Harvard	Fusformer	47.87 ± 5.13	2.84 ± 2.07	2.04 ± 0.99	0.986 ± 0.0101
Harvard	Fusion-reconstruction variant of MFGF-Net	43.80 ± 3.38	3.85 ± 0.62	3.10 ± 0.82	0.966 ± 0.0104
ARAD_1K	MFGF-Net	38.60 ± 1.85	4.75 ± 0.72	4.30 ± 0.86	0.965 ± 0.012
Agro-HSR	MFGF-Net	37.40 ± 2.05	5.35 ± 0.88	4.85 ± 0.92	0.948 ± 0.018

**Table 9:** Comparison of baseline models on the self-constructed wheat dataset.

Method	Input	Accuracy	Macro-F1	MAE
MFGF-Net	RGB + HSI	0.9689 ± 0.0225	0.9698 ± 0.0221	0.0593 ± 0.0429
SVM	HSI mean	0.8322 ± 0.0229	0.8328 ± 0.0245	0.1807 ± 0.0618
RF	RGB + HSI concat	0.8354 ± 0.0264	0.8360 ± 0.0301	0.1750 ± 0.0584
PLS-DA	HSI mean	0.7481 ± 0.0341	0.7143 ± 0.0479	0.6889 ± 0.0963
HybridSN	HSI	0.9448 ± 0.0159	0.9436 ± 0.0168	0.1917 ± 0.0482
1D-SSFTT	HSI	0.9560 ± 0.0108	0.9550 ± 0.0145	0.1630 ± 0.0417
ResNet18	RGB	0.4296 ± 0.0527	0.3948 ± 0.0641	1.2593 ± 0.1086
1D-CNN	HSI mean spectrum	0.8074 ± 0.0316	0.8094 ± 0.0349	0.5852 ± 0.0924

**Table 10:** Performance comparison of multimodal fusion models for identifying stages of natural mold spoilage in wheat.

Method	Characteristics	Accuracy	Macro-F1	MAE
MFGF-Net	Local Feature Fusion	0.9689 ± 0.0225	0.9698 ± 0.0221	0.0593 ± 0.0429
Concat + MLP	Shallow Feature Concatenation	0.9284 ± 0.0158	0.9271 ± 0.0164	0.2190 ± 0.0517
Global Cross-Attention	Global Cross-Modal Attention	0.9496 ± 0.0141	0.9487 ± 0.0148	0.1760 ± 0.0413
Transformer Fusion	Self-Attention + Cross-Attention	0.9581 ± 0.0127	0.9574 ± 0.0132	0.1180 ± 0.0336

**Table 11:** Paired *t*-test results between MFGF-Net and the top-performing baseline models based on three repeated runs (N=3, df=2).

Metric	Proposed MFGF-Net	Top Unimodal Baseline (1D-SSFTT)	Paired t-Test (vs. 1D-SSFTT)	Top Fusion Baseline (Transformer Fusion)	Paired t-Test (vs. Transformer Fusion)
Accuracy	0.9689 ± 0.0225	0.9560 ± 0.0108	t = 4.98, p = 0.038	0.9581 ± 0.0127	t = 5.24, p = 0.034
Macro-F1	0.9698 ± 0.0221	0.9550 ± 0.0145	t = 5.15, p = 0.036	0.9574 ± 0.0132	t = 5.81, p = 0.028
MAE	0.0593 ± 0.0429	0.1630 ± 0.0417	t = −7.43, p = 0.018	0.1180 ± 0.0336	t = −6.12,p = 0.026

**Table 12:** Performance comparison of different training objectives for wheat mold severity grading.

Experiment	Accuracy	Macro-F1	MAE
CE	0.9689	0.9698	0.0593
Ordinal	0.9185	0.8234	0.2440

**Table 13:** Ablation results for different modalities and fusion strategies.

Experiment	Accuracy	Macro-F1	MAE
MFGF-Net	0.9689 ± 0.0225	0.9698 ± 0.0221	0.0593 ± 0.0429
RGB-only	0.3481 ± 0.0462	0.2955 ± 0.0574	1.9260 ± 0.0231
HSI-only	0.8963 ± 0.0193	0.8936 ± 0.0199	0.3702 ± 0.0740
Simple Fusion Baseline	0.8667 ± 0.0261	0.8641 ± 0.0227	0.5263 ± 0.0713
Late Fusion Baseline	0.9309 ± 0.0171	0.9307 ± 0.0173	0.2395 ± 0.0556

**Table 14:** Ablation results for core modules.

Experiment	Accuracy	Macro-F1	MAE
MFGF-Net	0.9689 ± 0.0225	0.9698 ± 0.0221	0.0593 ± 0.0429
No MDCLA	0.8963 ± 0.0596	0.8949 ± 0.0501	0.3633 ± 0.0720
No FASR	0.9320 ± 0.0364	0.9290 ± 0.0107	0.3051 ± 0.0316
No Gating	0.9500 ± 0.0240	0.9493 ± 0.0131	0.1510 ± 0.0528

**Table 15:** Statistics of sample-level adaptive fusion weights.

Group	RGB	HSI	Cross-Modal
Overall mean	0.0078	0.0241	0.9682
Correct	0.0061	0.0216	0.9723
Incorrect	0.0434	0.0779	0.8787

## Data Availability

The data presented in this study are available from the corresponding author upon request.
